# Apyrase in horticultural crops: insights into growth, stress adaptation and quality regulation

**DOI:** 10.1186/s43897-025-00168-w

**Published:** 2025-09-01

**Authors:** Ying Li, Youxia Shan, Shuting Zhang, Jun Zhang, Junxian He, Hongxia Qu, Xuewu Duan, Yueming Jiang

**Affiliations:** 1 Guangdong Provincial Key Laboratory of Applied Botany, South China Botanical Garden, Chinese Academy of Sciences, Guangzhou, 510650 China; 2 College of Life Sciences, Gannan Normal University, Ganzhou, 341000 China; 3 School of Life Sciences and State Key Laboratory of Agrobiotechnology, The Chinese University of Hong Kong, Shatin, New Territories, Hong Kong, China; 4 University of Chinese Academy of Sciences, Beijing, 100039 China

**Keywords:** Apyrase, Energy, Extracellular ATP, Horticultural crops, Physiological activities, Signal transduction

## Abstract

Apyrases are a kind of nucleoside triphosphate diphosphohydrolases that catalyze the removal of the terminal phosphate group from nucleoside triphosphate (NTP) or nucleoside diphosphate (NDP). They also function either intracellularly or extracellularly in mediating the NTP/NDP homeostasis critical for plant growth, development, senescence, stress response and adaptation. Initial studies elucidated the biochemistry, structure and function of plant apyrases, while the recent progresses include the crystallography, newly discovered interaction partners and downstream targets for diverse apyrases. Furthermore, these apyrases play diverse roles in horticultural crops with the new recognition of extracellular ATP (eATP) receptors. This review summarized the types, structures, biochemical and physiological functions of plant apyrases and highlighted their roles in plant growth, development, biotic/abiotic stress responses and adaptation. The physiological activities among the apyrases, eATP with its receptor and eATP/iATP homeostasis, were reviewed. In particular, the quality formation / deterioration of postharvest horticultural crops caused by apyrases was emphasized. This paper reviewed the recent advances in the multiple roles of apyrases in horticultural crops and provided insights into the regulation of physiological activities by the enzyme from molecular network perspectives.

## Introduction

**Fig. 1:**
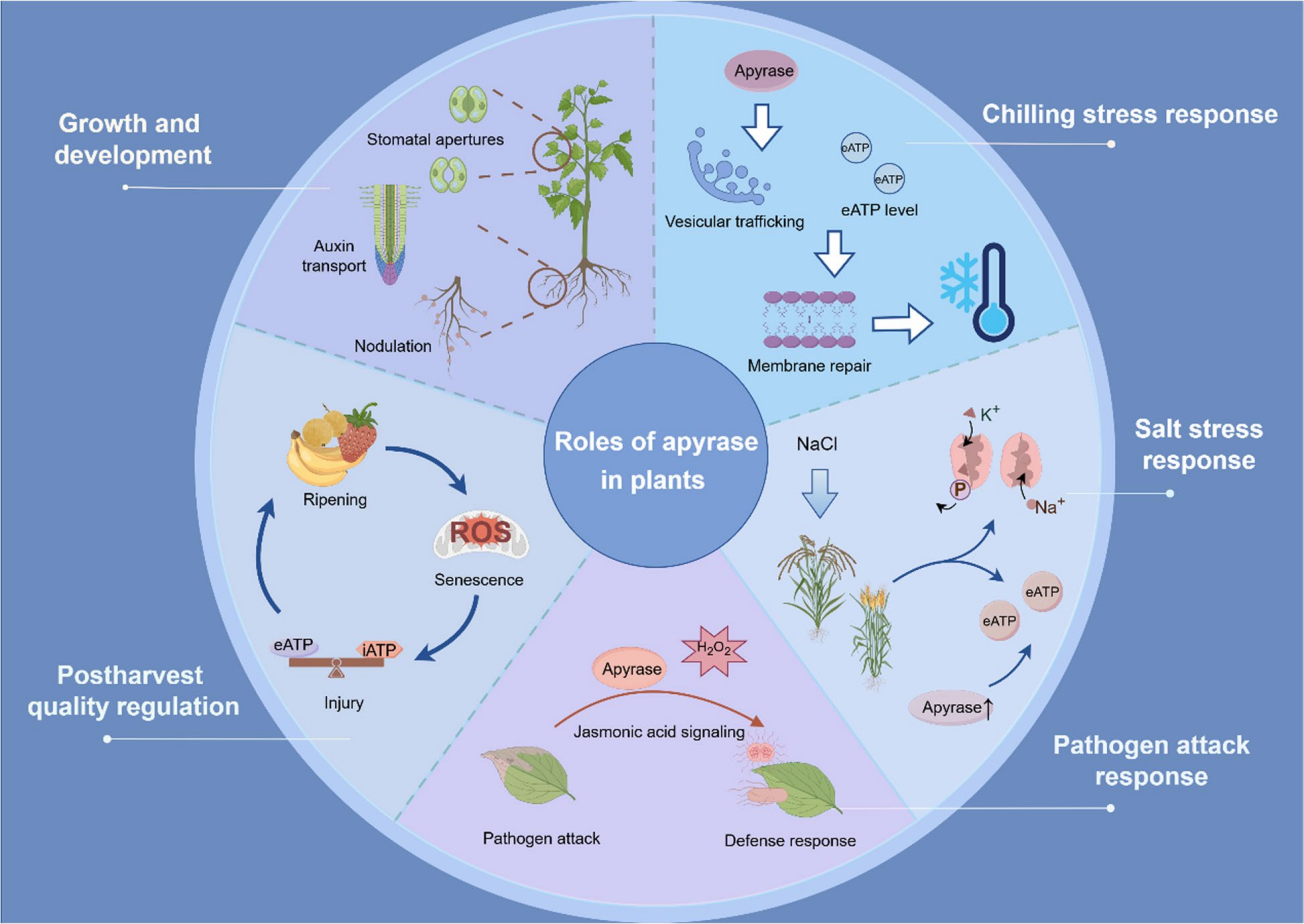
Overview of this review (by Figdraw). Apyrases play multiple roles in plants, such as the regulation of growth and development, abiotic stress response including chilling and salt stresses, biotic stress response including pathogen attack, and the regulation of postharvest quality

Energy is a key factor to trigger or maintain a series of physiological activities of living organism, including horticultural crops, while adenosine triphosphate (ATP) is established as the central source of energy to support cellular activities to mediate plant growth, development, senescence, stress response and adaptation (Deng et al. 2015). Based on cellular energy distribution, ATP is categorized into intracellular ATP (iATP) and extracellular ATP (eATP). While iATP as the energy supplier to support physiological activities can be released from the cytoplasm into the apoplast to become eATP via anion channels, gap junctions, ATP binding cassette transporters (ABCs) and vesicle exocytosis, the released eATP can act as a signaling molecule to mediate various physiological activities, such as plant growth, development, and responses to various biotic and abiotic stresses (Shan et al. 2022c). In energy metabolism, apyrase (APY) is the only kinase that can activate purinergic receptors, hydrolyze the terminal phosphate of nucleoside triphosphate (NTP) and nucleoside diphosphate (NDP) in the extracellular matrix and function either intracellularly or extracellularly to mediate NTP/NDP homeostasis, including iATP/eATP ratio. Apyrases ubiquitously exist in both prokaryotes and eukaryotes (Komoszyński and Wojtczak 1996). Plant apyrases belong to the E-type ATPase family and are firstly characterized in potato (*Solanum tuberosum*) (Krishnan 1955). In the last few decades, apyrases were reported in *Arabidopsis* (Chiu et al. 2015), pea (Hideaki Matsumoto et al. 1984), soybean (Day et al. 2000), cotton (Clark et al. 2009), wheat (Liu; et al. 2019), peanut (Sharif et al. 2023), banana (Shan et al. 2022a), populus (Deng et al. 2015) and rice (Tasnim et al. 2023). Furthermore, apyrases are divided into endo-apyrase and ecto-apyrase, and can exist in Golgi, endoplasmic reticulum and intracellular vesicles (Liu et al. 2019) and plasma membrane (Chiu et al. 2015). Previous investigation indicated that apyrases play important roles in a series of physiological processes, such as growth regulation of *Arabidopsis AtAPY1* by promoting auxin transport (Liu et al. 2012), cold tolerance of *Populus* by *PeAPY2* (Deng et al. 2015), responses of wheat to salt stress by *TaAPY7 (*Liu et al. 2019*)*, pea to pathogen attack by *PsAPY1* (Toyoda et al. 2016), and postharvest banana quality by *MaAPYs* (Shan et al. 2022a) (Table 1). With the increasing recognition of energy to mediate plant growth, development, senescence and stress response (Deng et al. 2015), and the new findings on the eATP receptor in *Arabidopsis* (Choi et al. 2014), the homeostasis of extracellular ATP (eATP)/intracellular ATP (iATP) may critically influence multiple physiological activities in horticultural crops. In particular, energy deficit is considered as a significant factor to influence postharvest quality and chilling tolerance in horticultural crops (Jiang et al. 2004; Wang et al. 2013). For instance, postharvest quality formation/deterioration of banana can be regulated by apyrases through eATP signal transduction or miRNA regulation (Shan et al. 2022a). Although the roles of energy metabolism in plant growth, development and stress response, as well as postharvest quality, have been widely investigated, how apyrases modulate these physiological and biochemical processes in relation to their mechanisms is less well-known. Furthermore, the functions and signaling pathways of plant apyrases reveal a significantly more intricate network than previously thought involved in the apyrases-mediated iATP/eATP homeostasis, Ca^2+^ signaling and reactive oxygen species (ROS). In this review, we summarized the types, structures, biochemical characteristics and physiological functions of plant apyrases and highlighted their roles in plant growth and development, senescence and biotic/abiotic stress response, with an emphasis with the postharvest quality formation/deterioration of horticultural crops (Fig. 1). This review aimed to provide insights into multiple roles of plant apyrases and discuss critically the recent advances in horticultural crops based on their multi-dimensional regulations from molecular network perspectives.

**Table 1:** Roles of APY genes in different species

	**Function**	**Species**	**Name of apyrase**	**Reference**
1	Growth and development	*Arabidopsis thaliana*	*AtAPY1; AtAPY2*	Steinebrunner et al. (2003)
			*AtAPY6; AtAPY7*	Yang et al. (2013)
		Pea (*Pisum sativum*)	*psNTP9*	Hideaki Matsumoto et al. (1984); Chen and Roux (1986)
		*Medicago truncatula*	*MtAPY1; MtAPY2*	Navarro-Gochicoa et al. (2003)
		Potato (Solanum tuberosum)	*StAPY2; StAPY3*	Riewe et al. (2008)
		Tuber mustard (*Brassica juncea* L.)	*BjAPY2*	Cao et al. (2015)
		Cotton (*Gossypium hirsutum*)	*GhAPY1; GhAPY2*	Clark et al. (2009)
2	Chilling response	Banana (*Musa acuminata* L.)	*MaAPYs*	Shan et al. (2022a)
		Strawberry (*Fragaria* × anannasa cv. Selva)	*APY1*	Aghdam et al. (2021)
		*Populus euphratica*	*PeAPY2*	Deng et al. (2015)
		Rice (*Oryza sativa*)	*OsAPY1; OsAPY2; OsAPY3*	Tasnim et al. (2023)
3	Salt stress response	Rice (*Oryza sativa*)	*OsAPY6*	Tasnim et al. (2023)
		Wheat (*Triticum aestivum*)	*TaAPY7*	Liu; et al. (2019)
4	Pathogen response	Pea (*Pisum sativum*)	*PsAPY1*	Toyoda et al. (2016)
		Peanut (*Arachis hypogaea* L.)	*AhAPY2 - 1*	Sharif et al. (2023)
		Pumpkin (*Cucurbita maxima*)	*Gene3360*	Zhao et al. (2022)
5	Postharvest quality	Edible mushroom (*Flammulina velutipes*)		Shi et al. (2020a)
		Strawberry (*Fragaria* × anannasa cv. Selva)	*APY1*	Aghdam et al. (2021)
		Banana (*Musa acuminata* L.)	*MaAPYs*	Shan et al. (2022a)

## Types and cellular locations of apyrases

**Fig. 2:**
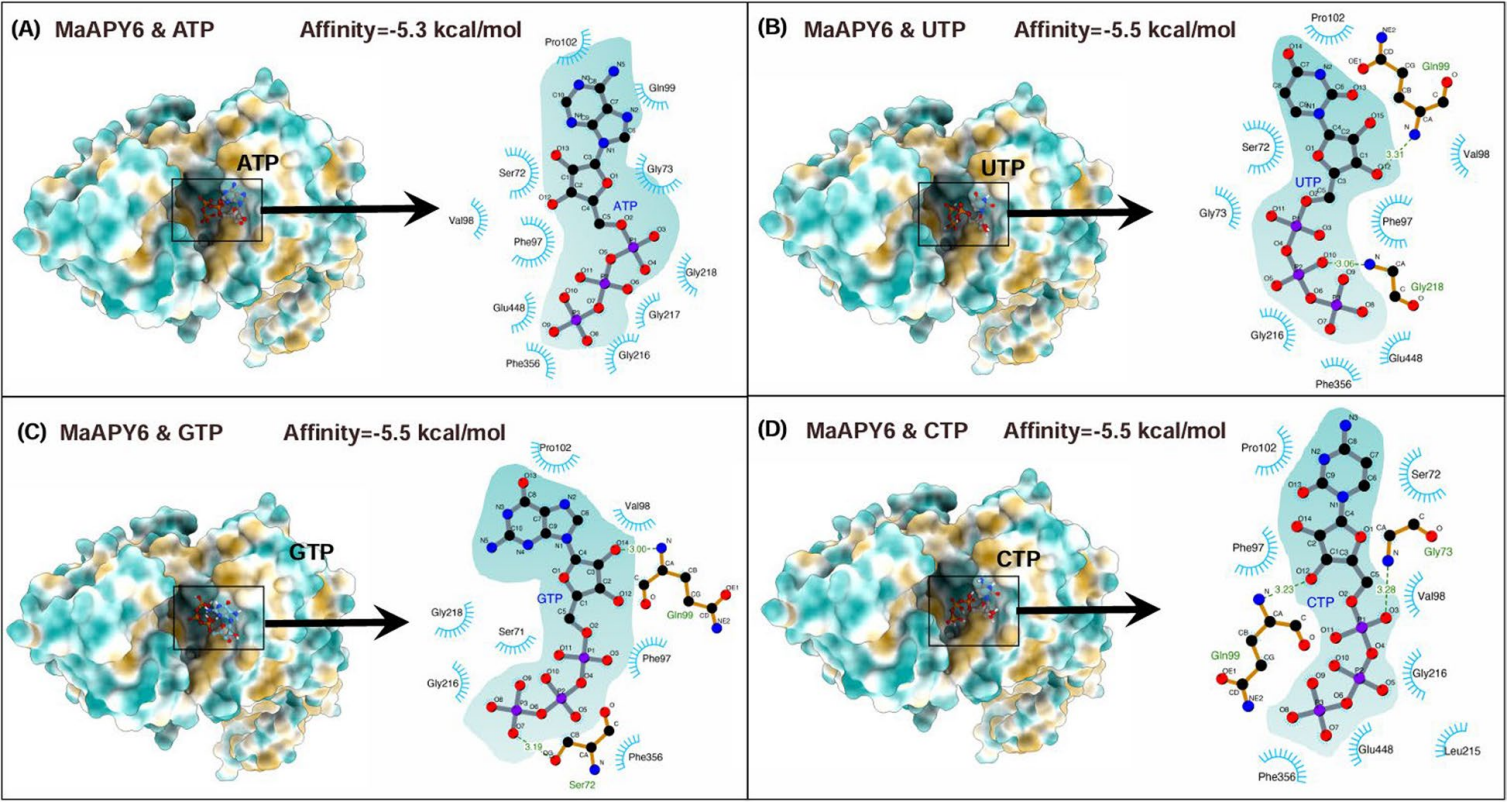
Schematic representation of the pivotal amino acid residues within banana MaAPY6 involved in the dephosphorylation of nucleoside triphosphates (NTPs) including ATP (**A**), UTP (**B**), GTP (**C**), and CTP (**D**). The precise three-dimensional structural model of MaAPY6 protein was derived from the trRosetta server through direct energy minimization by utilizing a constrained Rosetta version (Du et al. 2021). The NTP-MaAPY6 binding interaction was predicted using Autodock4.2 and Vina4 softwares (Trott and Olson 2010). The final outcomes were visualized using Chimera (Pettersen et al. 2004) and LigPLot (Laskowski and Swindells 2011)

Differed from other phosphatases, apyrases are the only kinase that can activate purinergic receptors and hydrolyze the terminal phosphate of nucleoside triphosphate and nucleoside diphosphate in the extracellular matrix (ECM) (Clark and Roux 2018). The enzyme is recognized as a member of guanosine diphosphatase 1 cluster of differentiation 39 (GDA1-CD39) superfamily (Knowles 2011) and plays the role in hydrolyzing nucleotides in the ECM of variety plants because it highly conserved throughout evolution (Clark et al. 2021). Furthermore, a characteristic feature of nucleoside triphosphate diphosphohydrolases (NTPDase) is the presence of the five apyrase conserved regions (ACRs) (Herbert et al. 2012), some of which belong to the β- and γ-phosphate-binding motifs of the actin/Hsp70/hexokinase superfamily (Tanaka et al. 2015). These APY structures have revealed similarities in the crucial ACR positions of catalytic cleft and the catalytic mechanism, as compared with other species (Summers et al. 2017). Especially, it seems that amino acids, such as phenylalanine, tryptophan and tyrosine, were well-conserved in all ACRs, which may be required for molecular interaction of apyrase (Clark et al. 2014). Different amino acid residues of apyrases in diverse species also influence their affinities and catalytic efficiencies on the five bases (A, G, C, T and U) of NTP and NDP. For example, AtAPY1 and soybean GS52 contain conserved glutamic acid residue, which is postulated to favor larger purines (NTPs) rather than small pyrimidines (NDPs) (Summers et al. 2017). The research conducted by Shan et al. (2022a, b) identified banana MaAPY6 as a crucial regulatory factor to involve in chilling injury in postharvest fruit. Herein, by the application of three-dimensional structural modeling and molecular docking techniques, the enzymatic active site ACR1 of MaAPY6 exhibited equivalent dephosphorylation capabilities for the four principal NTP molecules, with a comparable binding affinity of approximately 5.5 kcal/mol (Fig. 2), underscoring its remarkable efficiency in mediating NTP dephosphorylation. Thus, a further analysis of the amino acid sequence of apyrases is necessary to confirm how different structures affect their substrate affinities and specificities.

**Fig. 3:**
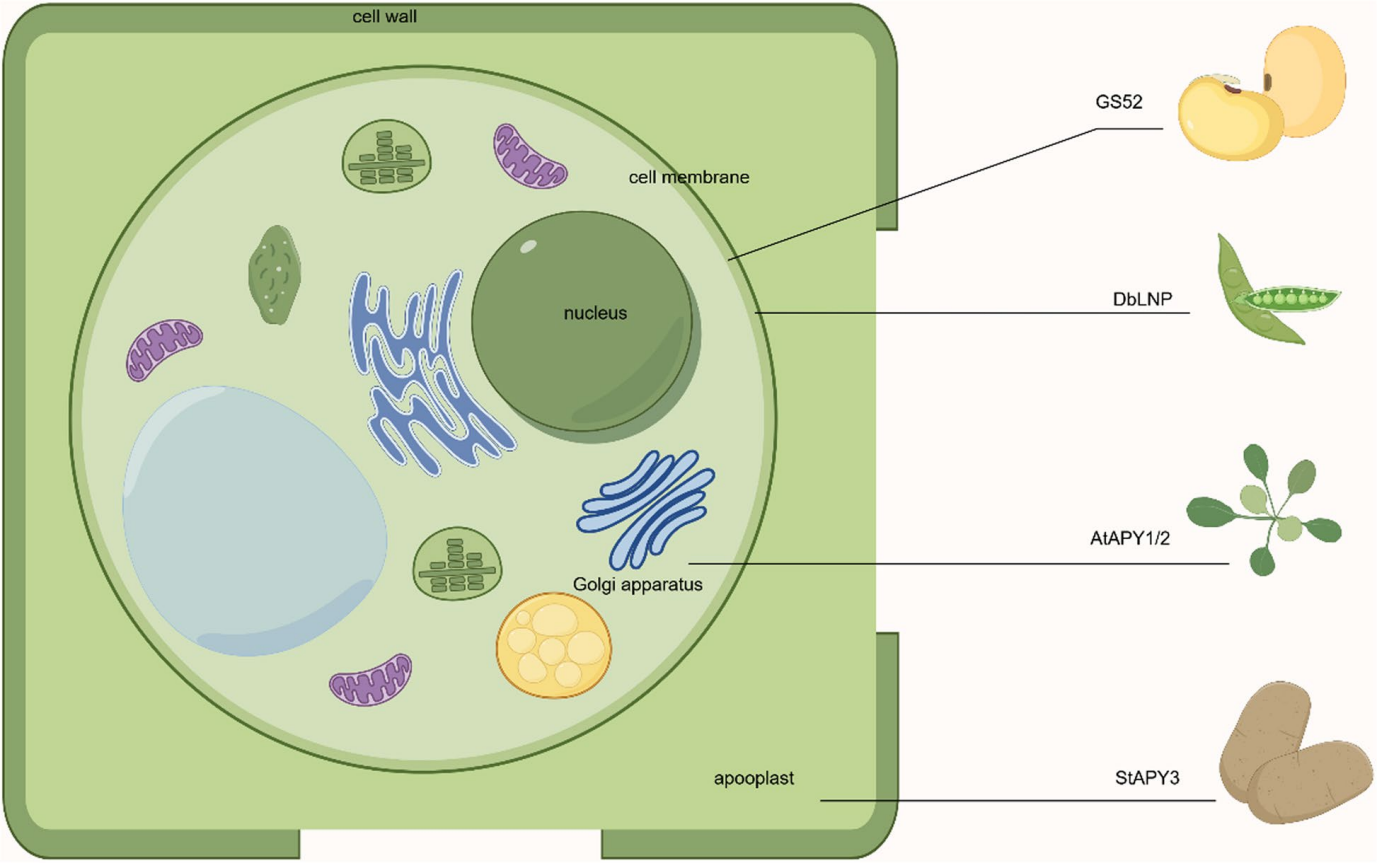
Differences in apyrase localization by Figdraw. Soybean (*Glycine soja*) apyrase GS52 and legume (*Dolichos biflorus*) apyrase named DbLNP were located in cell membrane (Etzler et al. 1999; Govindarajulu et al. 2009). AtAPY1/2 in *Arabidopsis* existed in the Glogi (Chiu et al. 2012), while potato (*Solanum tuberosum*) apyrase StAPY3 was located in the apoplast (Riewe et al. 2008)

**Fig. 4:**
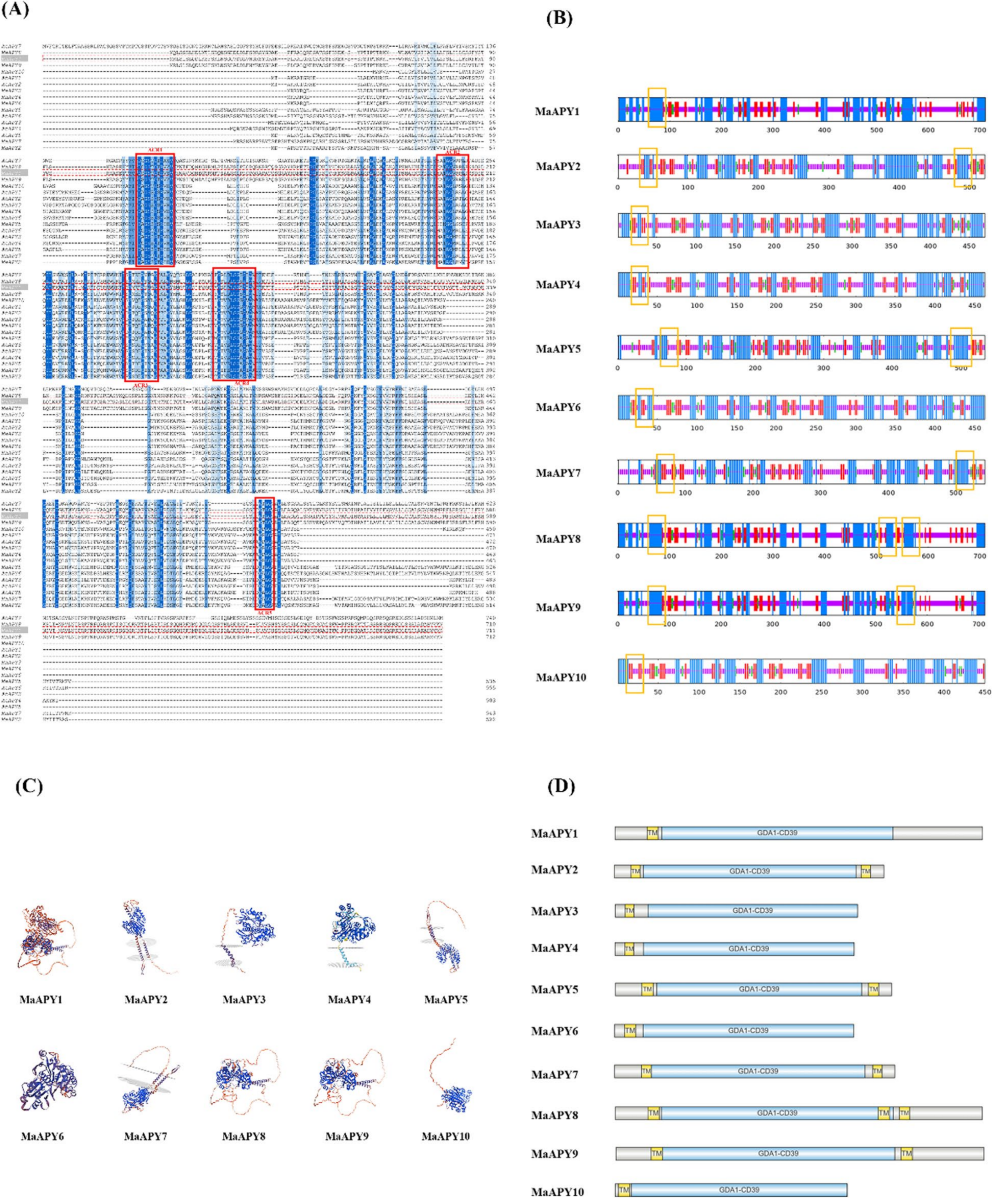
Secondary and tertiary structural analyses of the ten MaAPYs. **A** Full-length apyrase sequences of *Arabidopsis* and banana were aligned by jalviewg tool (Waterhouse et al. 2009). Five ACRs were labeled with red color box. **B** The secondary structure was made by submitting the amino acid sequence to the analysis tool NPS @ SOPMA (https://npsa-pbil.ibcp.fr/) (Deléage 2017). The corresponding membrane-spanning motif was identified by utilizing the online tool TMHMM server v.2.0 according to the default parameters (http://www.cbs.dtu.dk/services/TMHMM/) (Krogh et al. 2001) and then marked with a yellow color box. Alpha helix in blue, beta sheet in red, beta turn in green and random coil in purple were shown. C The structure models were constructed via the web server SWISS-MODEL (https://swissmodel.expasy.org/) (Waterhouse et al. 2018). **D** The relative sizes and locations of the GDA1-CD39 domains and transmembrane domains (TM) were visualized by IBS2.0 (https://ibs.renlab.org/) (Xie et al. 2022)

The first structure of plant NTPDase was elucidated from legume species *Trifolium repens* and *Vigna unguiculata* (Krishnan 1955). The conserved folding structure contains two modified RNAase-H folding motifs assembled from α-helical and β-sheet subdomains. These evidences suggested that NTPDases can adopt two conformations according to the molecule and co-factor bound in the active site (Summers et al. 2017). In contrast to ATPases, apyrases could collaborate with a variety of cofactors, including Ca^2+^, Mg^2+^, Mn^2+^ and Zn^2+^, while the ATPases only accompany with Mg^2+^ (Sharif et al. 2023). Transmembrane domains at the N- and C- terminals of plant apyrases feature amino acid glycosylation, which is require for the correct folding of proteins, targeting at the membrane, cellular distribution and enzymatic function (Clark et al. 2014). Based upon existing crystal structures, these studies of three-dimensional organization of plant apyrase are determined in potato (Kozakiewicz et al. 2008), *Arabidopsis* and pea (Clark et al. 2021). A transmembrane helix was reported on AtAPY1 protein with the globular portion as an ectodomain (Massalski et al. 2017). In contrast, potato APY showed a signal peptide rather than an inward-facing transmembrane helix, which could propose a different trafficking pathway compared with AtAPY1 (Kozakiewicz et al. 2008). As shown in Fig. 3, these differences appear to influence the localization of apyrases to regulate extracellular and/or intracellular ATP/ADP level. Therefore, it is worthwhile to further identify the tertiary structures of apyrases in other plant species. In banana fruit, 10 MaAPY proteins were determined based on the previous identification of *MaAPY* genes (Shan et al. 2022a) and all of 10 MaAPYs contained an N-terminal membrane-spanning motif (Fig. 4), which was similar with the APY proteins in wheat (Liu; et al. 2019) and rice (Tasnim et al. 2023). Taken together, the disparity of apyrases among different species may affect their localizations as well as the role in regulating extracellular and intracellular ATP/ADP levels or even iATP/eATP homeostasis.

## Roles of apyrase in energy metabolism/homeostasis

**Fig. 5:**
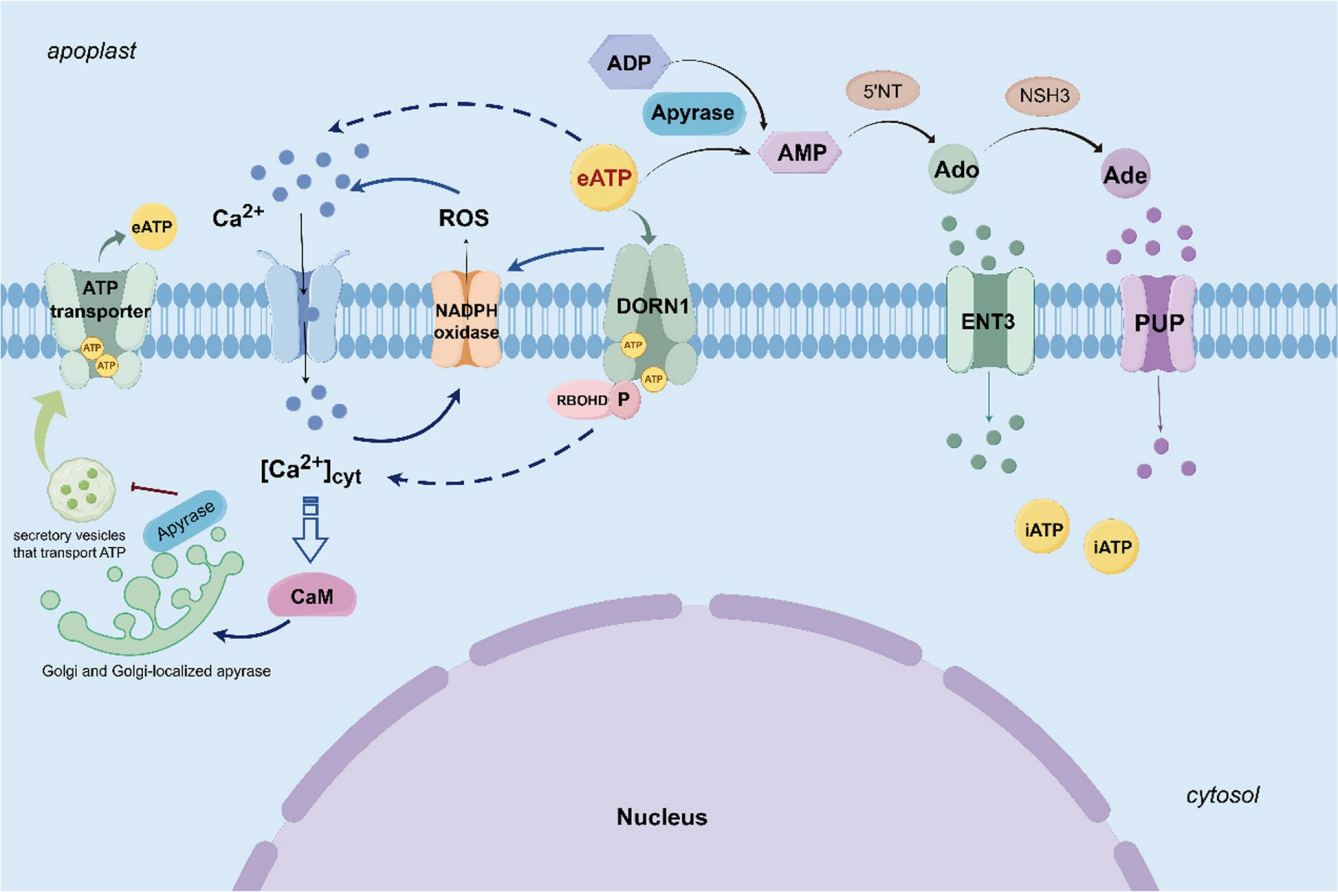
Overview of the molecular mechanisms of eATP/iATP homeostasis by apyrases by Figdraw. Under the basal condition, apyrases can catalyze the breakdown of ATP and adenosine diphosphate (ADP) to generate adenosine monophosphate (AMP). Subsequently, 5’-nucleotidase (5’NT) hydrolyzes further AMP to extracellular adenosine (eAdo). The latter can be brought into the cytoplasm by the equilibrative nucleoside transporter 3 (ENT3) or further catalyzed by the extracellular purine-specific nucleoside hydrolase 3 (NSH3) to remove sugar moiety and generate adenine (Ade) that could be transported into the cytoplast by a purine permease transporter (PUP). Apyrases in Golgi may decrease the eATP level by reducing the secretory vesicles to transport ATP to the ECM. Enzymatic activities of some apyrases could be enhanced by calmodulin, whose activities would increase when eATP generated. *DORN1* is regarded as the receptor for eATP, which could phosphorylate RBOHD and directly or indirectly involve in ROS and Ca^2+^ signaling pathways

Interestingly, although eATP can act as a crucial signaling molecule in various physiological activities, it is unable to move independently across the plasma membrane into the cytoplasm (Chivasa et al. 2011). Thus, it was proposed that eATP may be mediated by plasma membrane P2-type purinoceptors (Abbracchio et al. 2006). The first plant receptor of eATP was identified as a kinase that bound ATP with a high affinity in *Arabidopsis*, originally called Does Not Respond to Nucleotides 1 (*DORN1*) (Choi et al. 2014). Under the basal condition, apyrases can catalyze the breakdown of ATP and adenosine diphosphate (ADP) to generate adenosine monophosphate (AMP) (Wu et al. 2007; Tanaka et al. 2015). Subsequently, 5’-nucleotidase (5’NT) hydrolyzes further AMP to extracellular adenosine (eAdo). The latter can be brought into the cytoplasm by equilibrative nucleoside transporter 3 (ENT3) (Cornelius et al. 2012) or further catalyzed by extracellular purine-specific nucleoside hydrolase 3 (NSH3) (Jung et al. 2011) to remove sugar moiety and generate adenine (Ade) that could be transported into cytoplast by a purine permease transporter (PUP) (Moehlmann et al. 2014) (Fig. 5). Interestingly, Massalski et al. (2017) reported that apyrases preferred to ADP as a substrate, which provided a support for the hypothesis that AtAPY1 primarily functioned in Golgi (Chiu et al. 2012; Schiller et al. 2012). Similarly, a novel apyrase named MP67 localized in plasma membrane in *Mimosa pudica* exhibited a high substrate specificity for ADP, with a fourfold lower K_m_ value and 89-times higher activity for ADP than ATP (Okuhata et al. 2011). However, *Populus euphratica* apyrase (*PeAPY2*), unlike the APYs from *Arabidopsis* and potato, was more inclined to ATP as a substrate and insensitive to various ATPases (Windsor et al. 2003; Wu et al. 2007; Deng et al. 2015). Thus, it could not confirm enough that apyrases only recognize NTP or NDP as an enzymatic substrate.

The homeostasis of eATP/iATP is related to the release and transport of iATP, and the production and scavenging of eATP (Shan et al. 2022c). Generally, apyrases in ECM could directly affect eATP generation, but may decrease the eATP level in Golgi by reducing the secretory vesicles that transported ATP to the ECM (Clark et al. 2023) (Fig. 5**)**. As discussed above, hydrolytic eAdo and eAde could be retrieved to supply nucleotide and, thus, replenish iATP for energy supply in cells (Bernard et al. 2011; Sun et al. 2012; Wang et al. 2013; Feder et al. 2020). It is recognized generally that iATP is required for life maintenance and various physiological activities, such as plant growth, development and senescence, as well as responses to biotic and abiotic stresses (Sun et al. 2012). Sufficient iATP supply contributed to the resistance to cold stress by maintaining membrane integrity (Aghdam et al. 2018). In recent years, biochemical evidence has proved that eATP triggers a signaling cascade to serve as a hormone-like signal for physiological activities, such as plant development, gravitropism and defense response (Tang et al. 2003; Huang et al. 2012). In addition, eATP signaling is crucial for promoting iATP biosynthesis and reinforcing defense response by triggering signaling pathways of jasmonic and salicylic acids (Aghdam et al. 2018). Thus, to balance the iATP and eATP homeostasis for a series of physiological activities seems to be more importance in plants. Given the significant roles of eATP signaling, to explore further how apyrases impact exactly the downstream targets may help greatly to clarify the signal transduction chain.

## Roles of apyrase in growth and development

**Fig. 6:**
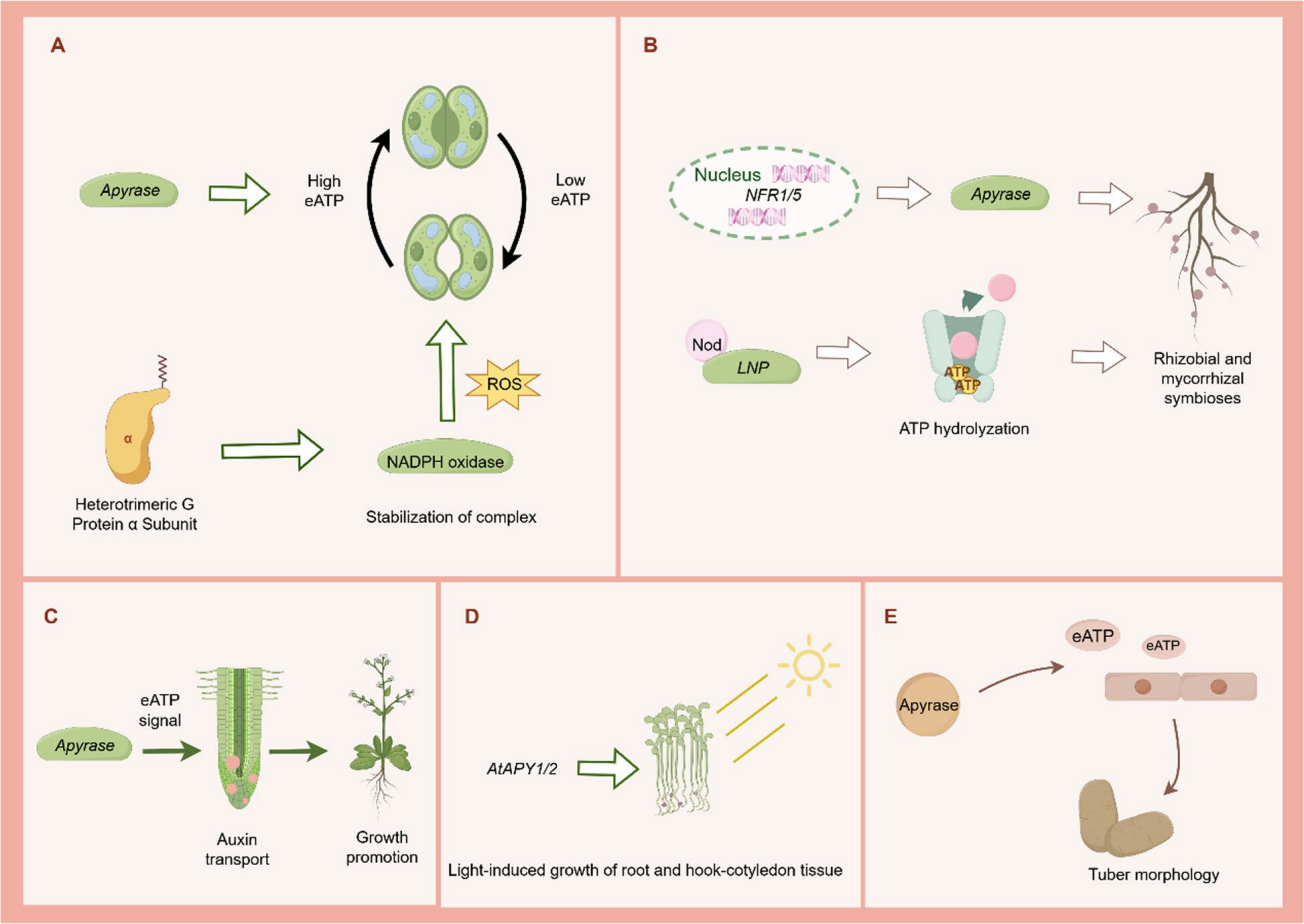
Roles of apyrases in plant growth and development by Figdraw. **A** Apyrase-mediated eATP to induce stomatal opening at low concentrations and stomatal closure at high concentrations. The heterotrimeric G protein may activate NADPH oxidase to connect ATP signaling with stomatal opening. **B** Apyrases may act as the downstream of Nod factor receptor (NFR) for both rhizobial and mycorrhizal symbioses. Lectin–nucleotide phosphohydrolase (LNP) contains a substrate specificity characteristic of apyrase and exhibits an ability to bind Nod factor for modulating the rhizobium–legume symbiosis. **C** Apyrases involve in polar auxin transport to regulate growth. **D** *AtAPY1/2* are necessary for the light-induced growth of root and hook-cotyledon of seedlings. **E** Apyrases affect tuber morphology by regulating the concentrations of eATP released by growing cells

As the Ca^2+^- and Mg^2+^-dependent NTPDases, apyrases can act as the key enzymes that restrict the concentration of extracellular nucleotides (Aghdam et al. 2018). The importance of apyrase to plant growth and development was initially discovered in pea as so-called psNTP9 (Hideaki Matsumoto et al. 1984; Chen and Roux 1986), with a molecular weight of approximately 47 kD and a K_m_ value of 0.6 mM for ATP (Chen et al. 1987). Other identified apyrases were *AtAPY1* and *AtAPY2*, which were not only located in EMC, but also in Golgi apparatus and cell nucleus in *Arabidopsis* (Chiu et al. 2012; Schiller et al. 2012). Apyrases affect phosphate absorption, leaf growth, root structure and seed size through diverse events in *Arabidopsis* and soybean (Veerappa et al. 2019). Pea apyrase contained a calmodulin-binding domain which allowed CaMs to combine apyrases and stimulate the physiological activities, thereby effectively transducing Ca^2+^ signals (Hsieh et al. 2000). Notably, *AtAPY1* could be induced by CaM, whose activities would increase when eATP induced (Clark and Roux 2018). With the exceptions of *AtAPY1* and *AtAPY2,* other members of *Arabidopsis* apyrases have been reported to regulate plant growth. For example, *AtAPY6* and *AtAPY7* affected exine development in pollen grains (Yang et al. 2013). Interestingly, leucine-rich repeat extensin (LRX) could combine the rapid alkalinization factor (RALF) and interact with the transmembrane receptor kinase FERONIA (FER) to regulate cell wall formation and cell growth (Dünser et al. 2019). Recent study reveals that *AtAPY7* appears to be a negative regulator of this LRX/RALF/FER signaling module that influences cell wall composition (Gupta et al. 2024). Additionally, apyrases were involved in polar auxin transport to regulate plant growth (Hammes et al. 2021). Polar auxin transport is an energy-consuming process that specifically regulates various critical growth and development processes throughout the plant’s life cycle, including embryonic development, organogenesis, lateral root morphogenesis, apical dominance and tropism (Semeradova et al. 2020; Hammes et al. 2021; Feng et al. 2022). As reported previously, a complete blocking of pollen germination and severe growth inhibition occurred in double-knockout mutations of *AtAPY1* and *AtAPY2* (Steinebrunner et al. 2003; Chiu et al. 2015). On the contrary, overexpression of either *AtAPY1* or *AtAPY2* in *Arabidopsis* could promote plant growth and auxin transport (Wu et al. 2007; Liu et al. 2012) (Fig. 6C). Elevated ATP levels or inhibited expression of apyrase gene suppressed basipetal auxin transport and limited cell elongation and mitosis (Liu et al. 2012). It was noted also that APY1 may affect the absorption of phosphate by directly increasing the extracellular enzymatic activity and releasing Pi, as well as indirectly regulating eATP level and signal transduction, including purinergic receptor signaling and auxin response (Slocum et al. 2023). Recently, annexins have been determined to participate in apyrase- and eATP-mediated auxin transduction. *AtANN3* was found to affect the eATP-regulated seedling growth by modulating polar localization of auxin transporters (Xu et al. 2023). Thus, apyrases may potentially modulate the growth speed of plant cells as eATP regulators, which needs to be further elucidated.

Apyrases can regulate leaf functions during plant development by influencing photosynthesis and stomatal aperture. Wu et al. (2007) reported that the transcripts and protein levels of *AtAPY1/2* were necessary for light-induced growth of root and hook-cotyledon tissue of seedlings (Weeraratne et al. 2022; Clark et al. 2023) (Fig. 6D). The suppression of apyrase activity reduced the red-light-induced growth of primary roots, apical hooks and cotyledons (Weeraratne et al. 2022), in agreement with the report of Wu et al. (2007). The eATP signaling pathway exhibits also other effects on the growth and development of aerial tissues in *Arabidopsis*. For example, application of 1 mM ATP enhanced Ca^2+^- and H_2_O_2_-dependent photosystem II (PSII) photochemistry in leaf of kidney-bean (*Phaseolus vulgaris*) (Feng et al. 2015). The differential expressions of the pathogen defense-related proteins and photosynthesis of tobacco leaf were induced by ATP treatment (Shi et al. 2020b). Based on the findings that *AtAPY1* could be regulated by light (Wu et al. 2007), further investigation should be performed to reveal how APY participates in the light-regulated pathway to affect plant growth and development.

Stomatal closure is a central response to affect plant growth and development. It has been assumed that the changes in stomatal apertures were accompanied by exocytosis- and endocytosis-mediated plasma membrane stretching and shrinkage (Shope et al. 2003). Previous investigations have proposed that the activities of *AtAPY1* and *AtAPY2* mediated the eATP level in *Arabidopsis* (Tang et al. 2003). Applications of ATPγS and ADPgS regulated the eATP levels and generated the biphasic effect on stomatal apertures, with opening at low concentrations and closure at high concentrations (Fig. 6A). Another report exhibited that cotton fiber apyrases *GhAPY1* and *GhAPY2,* highly similar to *AtAPY1* and *AtAPY2,* involved in maintaining an optimal eATP level during the rapid phase of fiber elongation (Clark et al. 2009). Such effect can be improved also by applying a low concentration of ATPγS or ADPgS. Specifically, ROS participates in the eATP-induced stomatal movement. *DORN1* is regarded as the receptor for the eATP-induced stomatal closure of guard cells, followed by directly phosphorylating RBOHD and elevating production of ROS (Chen et al. 2017). *Populus euphratica* apyrases *PeAPY1* or *PeAPY2* overexpressed in *Arabidopsis* also increased the ABA action in the eATP-induced stomatal control, while in transgenic plants, ABA enhanced the transcriptions of *AtRBOHF* and *AtRBOHD*, leading to more significant stomatal closure and water-retaining capacity (Zhang et al. 2021a). Besides, investigation of Ca^2+^ influx in eATP signal transduction in guard cells demonstrated that heterotrimeric G protein may promote stomatal opening with ROS-related ion transport (Hao et al. 2012) (Fig. 6A).

In the aspect of tuber morphology, Riewe et al. (2008) proposed the hypothesis that changes of potato-specific apyrase expression affected plant development by influencing eATP signaling transmission (Fig. 6E). Likewise, *BjAPY2* was reported to involve in the stem expansion in tuber mustard (*Brassica juncea* L.) by regulating the concentration of the eATP released (Cao et al. 2015). Additionally, in terms of root development, apyrases were postulated to act the downstream of the nodule development, such as Nod factor receptor 1 (NFR1) and Nod factor receptor 5 (NFR5) (Broghammer et al. 2012), which was essential for both rhizobial and mycorrhizal symbioses (Tanaka et al. 2015) (Fig. 6B). In the case of soybean, the GS52 apyrase possessed a catalytic domain required for stimulation of enhance nodulation (Tanaka et al. 2011). On the other hand, Nod factors produced by rhizobial strains could stimulate ATP hydrolyzation, which could interact with the lectin nucleotide phosphohydrolase (LNP), known to a novel class of Nod factor receptor with apyrase activity in *Dolichos biflorus* (Etzler et al. 1999). However, apyrase-like genes are not induced in response to Nod factor or regulated by rhizobia in *M. truncatula*, but induced rapidly and transiently by rhizobial stress. Therefore, further investigation is warranted to confirm whether apyrases directly regulate the symbiosis in plant growth and development.

## Response of apyrase to plant chilling stress

**Fig. 7:**
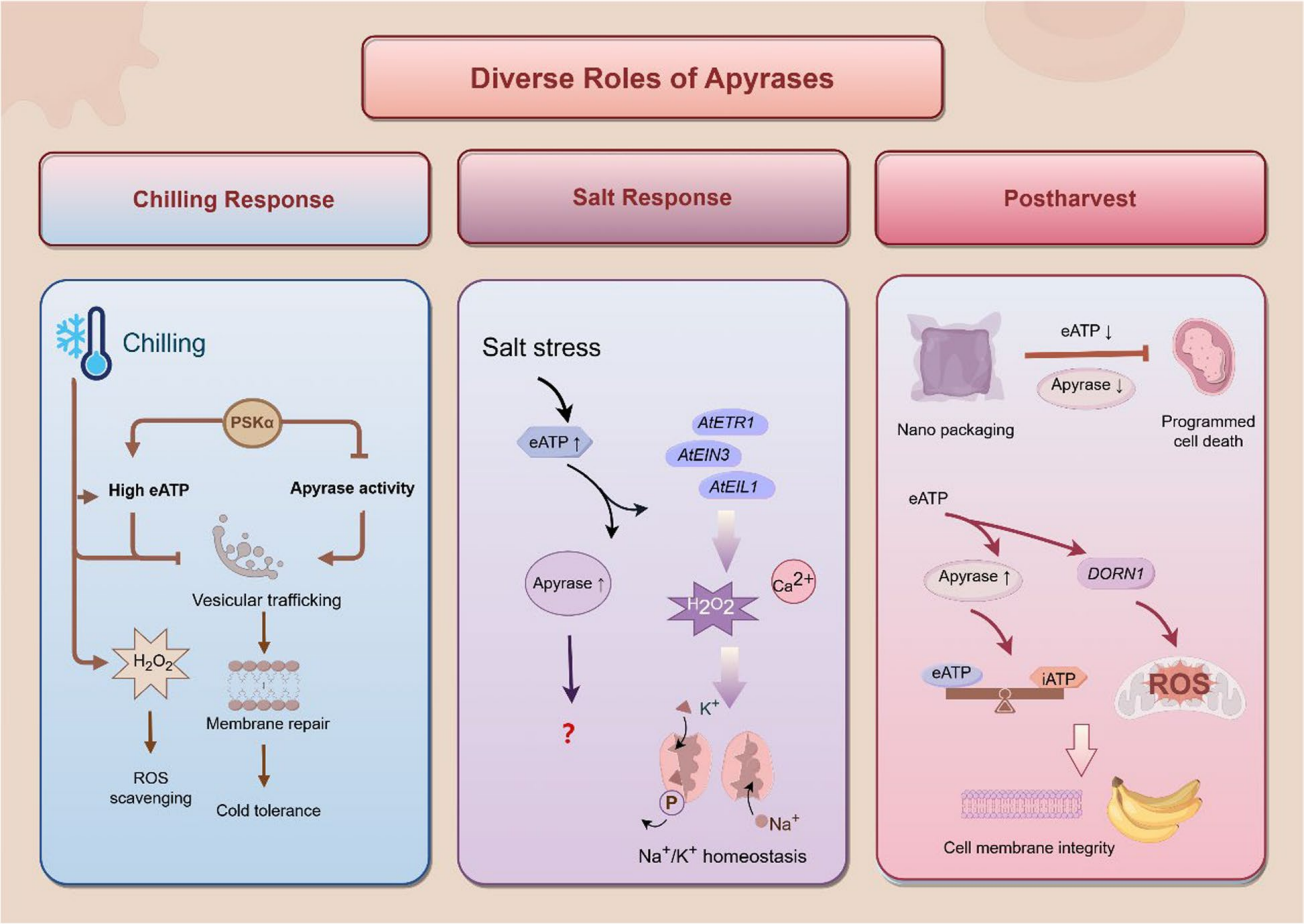
Roles of apyrases in chilling tolerance, salt stress response and postharvest quality regulation by Figdraw. The treatment with phytosulfokine α (PSKα) contributed to reduction of apyrase expression and increase of eATP level. Apyrases could accelerate the vesicular trafficking to increase membrane repair and avoid the inhibition of vesicular trafficking by cold-induced ATP accumulation. The expressions of APY genes were upregulated by NaCl treatment. Salt-elicited eATP triggered the transcriptions of ethylene-related genes, such as *AtEIN3*, *AtEIL1* and *AtETR1,* which further mediated H_2_O_2_ and cytosolic Ca^2+^ signaling cascades to regulate K^+^/Na^+^ homeostasis. In terms of postharvest quality, nano packaging was beneficial for delaying programmed cell death (PCD) during storage by inhibiting eATP increase and elevating apyrase activity. eATP induced *DORN1* expression to regulate ROS while apyrases involved in the iATP/eATP homeostasis, which could ensure cell membrane integrity for improving cold tolerance. Arrows represented promotion, and termination symbols represented suppression. *EIN3*, Ethylene insensitive 3; *ETR1*, Ethylene response 1; *EIL1*, Ethylene insensitive-like 1; *DORN1*, Does Not Respond to Nucleotides1

Plants rapidly respond to chilling stress, including a series of changes in the membrane fluidity along with a high electrolyte leakage, accumulation of ROS, fluctuation of [Ca^2+^]_cyt_, alteration of gene expression and reprogramming of transcriptomic or metabolomic signatures (Jiang et al. 2004). Particularly, chilling is one of abiotic stresses involved in the Ca^2+^ signaling and the mediated-eATP/iATP homeostasis (Shan et al. 2022a). eATP involved also in diverse interactions between plant hormone and signaling pathway in abiotic stress responses (Lang et al. 2017). Overexpression of *PeAPY2* in *Arabidopsis* improved the vesicular structure and regulated the eATP level for preventing the inhibition of the vesicular trafficking and membrane repair by ATP (Fig. 7), which conferred the cold tolerance of *Populus euphratica* (Deng et al. 2015). Furthermore, application of phytosulfokine α (PSKα) could delay senescence by triggering eATP signaling during cold storage in strawberry fruit, while the lower *APY1* expression but higher eATP level in the fruit treated with 150 nM PSKα supposed to induce NADPH oxidase activity, then promote ROS scavenging ability, and, finally, reduce chilling symptoms (Aghdam et al. 2021) (Fig. 7). In rice, although the expression levels of *OsAPY1*, *OsAPY2* and *OsAPY3* significantly increased during chilling stress (Tasnim et al. 2023), the possibility in regulating the ATP level needs to be confirmed further. In terms of energy metabolism, newly discovered interaction partner and downstream signaling transduction for diverse apyrases will help to elucidate better the mechanism of chilling injury. In addition, it has been found that micro RNAs (miRNAs) as a class of negative regulatory molecules could suppress their target genes via guiding mRNA degradation and/or translational repression (Kong et al. 2025), while such as miRNA159 and miRNA319 may target and alter the expressions of apyrases in banana fruit involved in postharvest chilling injury (Shan et al. 2022a),. However, the detailed physiological mechanisms for these interaction factors of apyrases require to be investigated further.

## Response of apyrase to salt response

Salt stress mainly occurs in osmotic or ionic way, resulting from reduction of water potential and disruption of ions uptake (Munns et al. 2020; Paheli and Debasis 2021). Among these salt stresses, the accumulation of sodium chloride in irrigated soil is a common issue to inhibit plant growth and development (Julia et al. 2019). The flow of Na^+^ and the synthesis of organic solutes under salt stress condition consume a mass of energy (Yan et al. 2021). In vivo assay suggested that salt stress inhibited plant growth by releasing eATP and activating purinergic signaling (Daewon et al. 2023). In rice, RNA-seq data showed that *OsAPY6* may be the potential gene in response to salt stress (Tasnim et al. 2023). The genome-wide identification and expression pattern of apyrases in wheat indicated that a total of nine *APY*s were upregulated after 300 mM NaCl treatment and, specially, *TaAPY7* plays a pivotal role in response to salt stress (Liu et al. 2019). The released eATP triggered the transcriptions of the ethylene-related genes in *Arabidopsis thaliana*, such as *AtEIN3*, *AtEIL1* and *AtETR1*, and then induced H_2_O_2_ and Ca^2+^ signaling pathways to regulate K^+^/Na^+^ homeostasis under salt stress condition (Tao et al. 2020). These results provide interactive effects of eATP, ethylene and [Ca^2+^]_cyt_ to maintain K^+^/Na^+^ homeostasis and suggest a potential role of apyrases to act together with plant hormone in regulating salt stress response (Fig. 7).

## Response of apyrase to pathogen attack

**Fig. 8:**
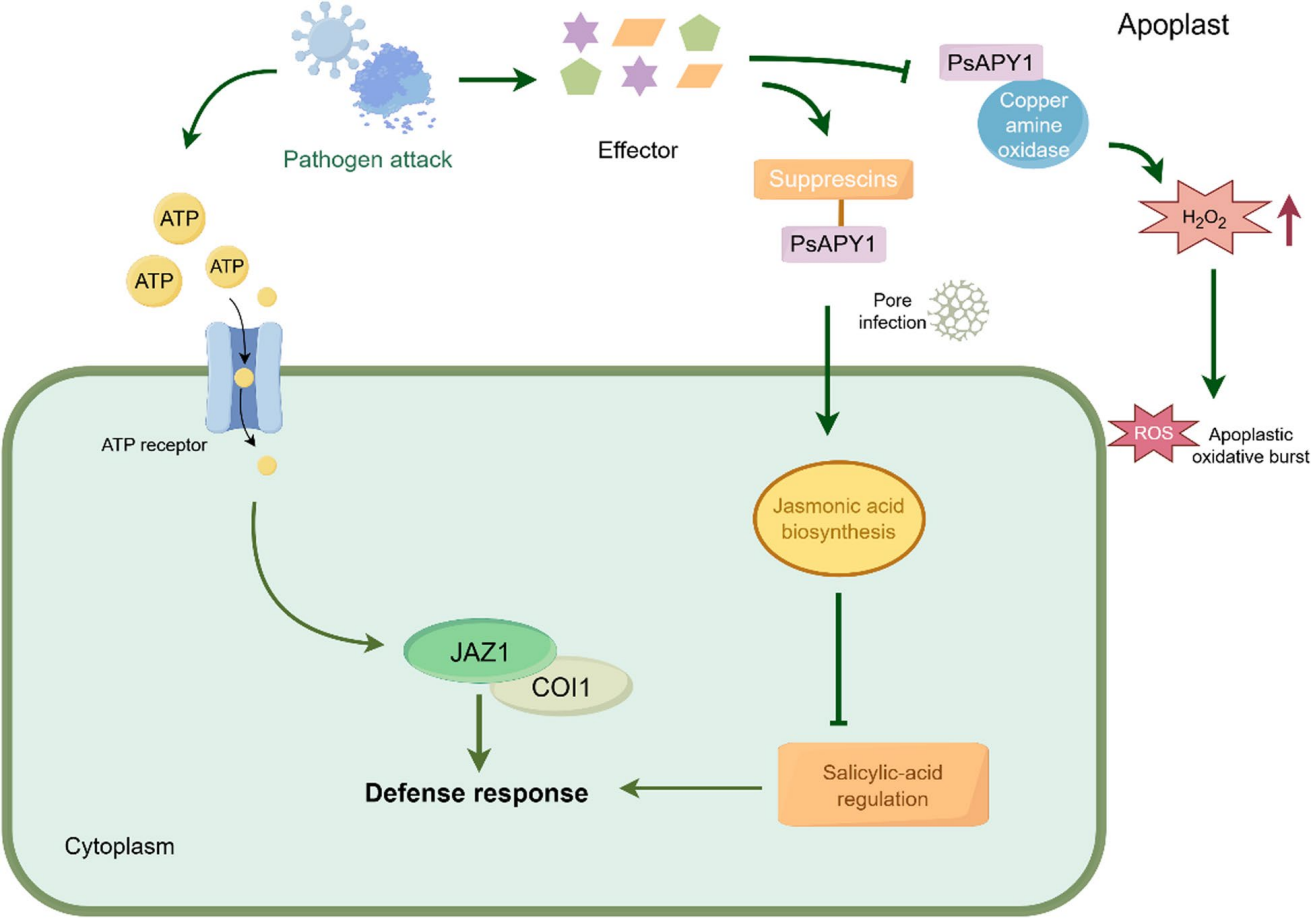
Roles of apyrases in pathogen attack response by Figdraw. The release of eATP caused by cellular damage after pathogen infection could be recognized by the eATP receptor to enhance the interaction between jasmonate ZIM-domain (JAZ) and coronatine-insensitive1 (COI1) proteasome and then to increase plant defense systems. Pathogenic fungi could target ecto-ATPase (PsAPY1) and achieve spore infection by secreting supprescins, an extracellular effector. On one hand, these supprescins participated in jasmonic acid (JA)-mediated signaling to attenuate salicylic acid (SA)-regulated defense. On the other hand, supprescins could inhibit the event that *PsAPY1* initiated the apoplastic oxidative burst such as H_2_O_2_ production by copper amine oxidase and then affect pathogenic susceptibility. Arrows represented promotion, and termination symbols represented suppression

Pathogen-associated molecular pattern (PAMP) is established for plants to respond to various pathogens (Cao et al. 2014; Giulia and Felice 2022). The secreted pathogen-derived effectors can achieve infection by the suppression or circumvention of the PAMP-triggered immunity (PTI) before or during infection process (Jones and Dangl 2006). Furthermore, peanut *AhAPY2 - 1* could modulate foreign gene expression in a pericarp-abundant manner for pathogen response (Sharif et al. 2023), while pumpkin (*Cucurbita maxima*) apyrase-like gene 3360 plays an important role in the resistance to the gummy stem blight (GSB) caused by *Stagonosporopsis cucurbitacearum* infection (Zhao et al. 2022), which is referentially valuable for the research of apyrase in pathogen response. On the other hand, a number of pathogenic fungi could attack plants by restraining apyrase activities. For example, a successful infection by a well-known pathogenic fungus in pea (*Mycosphaerella pinodes*) can be achieved by secreting extracellular effectors, such as supprescins, to target *PsAPY1* and then cause a monomerization, which was beneficial for spore infection by antagonizing salicylic acid regulation and elicitor-induced defense at the early stage of infection (Toyoda et al. 2016). Thus, *PsAPY1* appears to recognize pathogen-derived elicitors and may directly interact with co-localized copper-containing amine oxidase that produced H_2_O_2_, eventually initiating or maintaining the apoplastic oxidative burst (Toyoda et al. 2012; Toyoda et al. 2016) (Fig. 8). For fungal response, only 2 apyrases in *Saccharomyces cerevisiae*, named as *GDA1* and *TND1* (Gao et al. 1999), were both Golgi-localized and indispensable for glycosylation (Abeijon et al. 1993). Subsequently, GDA1 or TND1 gene has been identified in *Candida albicans* (Herrero et al. 2002), *Sporothrix schenckii* (López-Esparza et al. 2013) and *Kluyveromyces lactis* (Uccelletti et al. 2007). An xenobiotic efflux model mediated by co-symport of toxin and ATP via P-glycoprotein in yeast and *Arabidopsis* suggested that a low steady-state level of eATP mediated by apyrase is necessary for sustaining the efflux of xenobiotics (Thomas et al. 2000).

It is noted that the cross-talk between plant hormones and eATP, and the increases in the [Ca^2+^]_cyt_ and ROS levels can regulate the DAMP immune responses (Clark and Roux 2018). *DORN1* mediated the eATP level as a crucial signaling role in plant defense (Choi et al. 2014). For example, *DORN1* impacted jasmonic acid signaling and promoted the resistance to pathogens (Claudine et al. 2017). In *Arabidopsis*, eATP induced the resistance against necrotrophic fungus, *Botrytis cinerea*, by reducing jasmonate ZIM-domain (JAZ) protein stability via triggering coronatine-insensitive1 (COI1) proteasome pathway through Ca^2+^ ROS, NO, etc. (Fig. 8). This event can be enhanced in the *DORN1* over-expression line (Tripathi et al. 2018). Taken together, the direct crosstalk between eATP and JA signal pathway has provided an insight into plant defense responses.

## Roles of apyrase in postharvest quality

Postharvest horticultural crops undergo a series of physiological activities, including maturation/ripening, senescence and quality formation/deterioration. Specially, the most important quality traits, such as sensory appearance and nutritional compositions of horticultural crops, are due to dramatically metabolic processes during storage. Energy is a control center to trigger maturation/ripening, senescence or quality formation/deterioration of postharvest horticultural crops during storage (Jiang et al. 2007; Aghdam et al. 2018). For instance, the browning of litchi pericarp was correlated with cell membrane damage caused by energy depletion (Duan et al. 2005; Zhou et al. 2022). To date, ATP treatment extended the shelf life of tulip (Azad et al. 2008), carnation flowers (Song et al. 2014), longan (Chen et al. 2015), litchi (Yi et al. 2009) and banana (Shan et al. 2022b), illustrating that sufficient iATP and high ATP and ADP levels can delay the programmed cell death (PCD) during postharvest storage. Interestingly, edible mushroom PCD with nano-packaging can be delayed during storage through inhibiting eATP amount and elevating apyrase activity (Shi et al. 2020a) (Fig. 7). Excessive ROS accumulation may lead to the damage and dysfunction of some important enzymes during plant senescence (Rey and Tarrago 2018). Treatment with 150 nM PSKα contributed to low APY1 expressions with high eATP levels and H_2_O_2_ accumulation, while H_2_O_2_ to serve as a signaling molecule may further induce a high SUMO E3 ligase (SIZ1) expression, which involved in the sumoylaltion for regulating protein stability (Ghimire et al. 2020; Souleimen and Laurent 2021) and accounted for senescence delay and decay control in strawberry fruit (Aghdam et al. 2021).

It is reported that the apyrase-mediated eATP specifically induced the expression of *MaDORN1.19* to enhance cold tolerance of postharvest banana fruit during storage (Shan et al. 2020, 2022b). Subsequently, they identified a total of ten banana APYs genes and found that application of 1 mM ATP increased the transcription level of *MaAPYs* and maintained relatively the iATP/eATP homeostasis, which indicated that the apyrase-mediated eATP signal in reducing chilling injury of banana fruit during storage (Shan et al. 2022a) (Fig. 7). These results highlighted the vital functions of eATP in response to chilling temperature in fresh horticultural crops (Aghdam et al. 2018). Nevertheless, the regulation of postharvest quality by apyrases is mainly reflected in eATP signaling molecule in horticultural crops. Besides, apyrases may have different mechanisms involved in a series of physiological activities, such as ripening, senescence and quality formation or deterioration, with an emphasis on the synergistic effects at multiple physiological activities and across multiple genes. In the future, to combine proteomic and metabolic technologies will help to elucidate comprehensive regulatory networks caused by apyrases or the ATP-related genes and then improve further postharvest technology of horticultural crops.

## Conclusion and future prospects

**Fig. 9:**
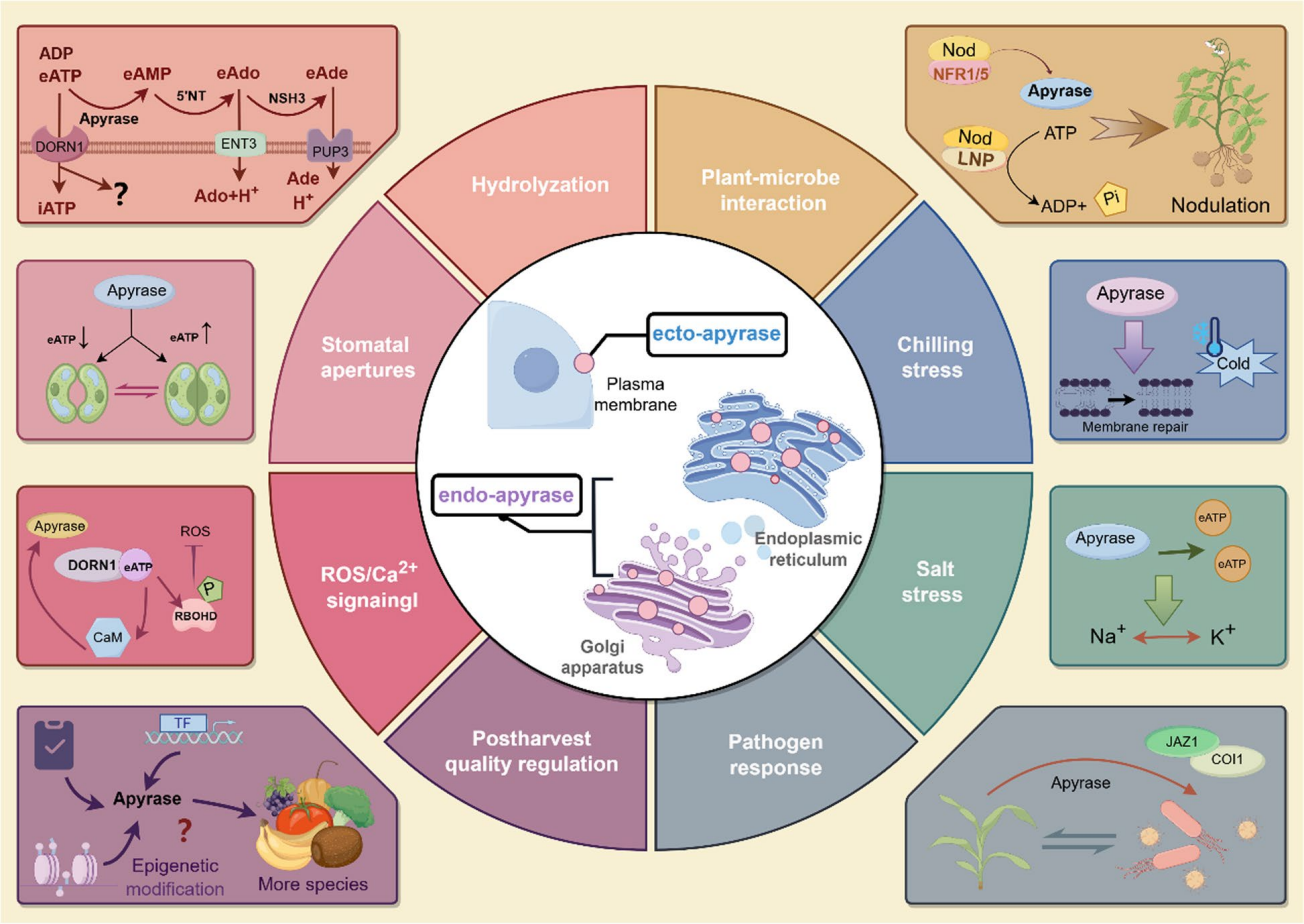
Multiple functions of plant apyrase by Figdraw. 5’NT, 5’-nucleotidases; Ade, Adenine; Ado, Adenosine; ADP, Adenosine diphosphate; AMP, Adenosine monophosphate; CaM, Calmodulin; COI1, Coronatine-insensitive1; DORN1, Does Not Respond to Nucleotides1; eAde, Extracellular adenine; eAdo, Extracellular adenosine; eATP, Extracellular ATP; ENT3, Nucleoside transporter 3; iATP, Intracellular ATP; JAZ1, Jasmonate ZIM-domain 1; LNP, Lectin nucleotide phosphohydrolase; NDP, Nucleoside diphosphate; NFR, Nod factor receptor; NSH3, Nucleoside hydrolase 3; ROS, Reactive oxygen species

Significant progresses in our understanding of the multiple roles in plant apyrases have been achieved over the last decade, but the detailed signaling pathway or metabolism network of the apyrases in relation to eATP remains to be clarified in the future. For example, the number of apyrases vary in different species, but their functions remain to be determined further. Among the 7 apyrases of *Arabidopsis*, *AtAPY1* and *AtAPY2* as the well-characterized genes, modulated plant growth (Carolin et al. 2007; Meng et al. 2019). Furthermore, among these 13 different soybean apyrases only GS52 was identified as a plasma membrane-associated ectoapyrase (Day et al. 2000; Govindarajulu et al. 2009). There were 10 apyrases in potato species, but only 3 *APY*s that really worked in ECM have been verified so far (Riewe et al. 2008). Unfortunately, the APY genes in other horticultural species remain relatively unknown. For the potential application, it is necessary to elucidate apyrases how to affect the regulation of energy system, eATP signaling functions and downstream genes to improve the quality traits and stress resistance of horticultural crops. In this respect, genome-wide identification, expression pattern and metabolism network will help greatly to understand better the multiple roles of apyrases in the iATP/eATP homeostasis in relation to a series of physiological activities (Zhu et al. 2022). In addition, more investigations, such as posttranslational modification and involvement of non-coding RNAs (ncRNAs), could be performed to elucidate the underlying multiple roles of apyrase in growth, stress, adaptation and quality of horticultural crops (Zhang et al. 2021b; Chen et al. 2024). Although a number of researches proved that eATP acts as a signal molecule involved in physiological activities, how APY carries out its functions in the metabolism network remains to be elucidated. There are more challenges to relate to the interplay between eATP with its receptor and signaling pathways, such as ROS, NO and Ca^2+^, while how the generation of eATP and the eATP/iATP homeostasis trigger these physiological activities remains to be understood well. More attention should be paid to not only the production of eATP with energy regulation, but also downstream biochemical reactions and physiological pathways. In conclusion, this review summarizes the recent progress in multiple roles of apyrases in plant growth, development, responses to abiotic stress and pathogen, and postharvest quality, and provides insights into the regulation of physiological activities by the apyrases from molecular network perspectives, as shown in Fig. 9.

## Data Availability

Not applicable.

## Abbreviations

5’NT: 5’ Nucleotidases
7 WC: *Trifolium* *repens*
ABCs: ATP binding cassette transporters
ACRs: Apyrase conserved regions
Ade: Adenine
Ado: Adenosine
ADP: Adenosine diphosphate
AMP: Adenosine monophosphate
ANN: Annexins
APY: Apyrase
*AtEIL1*: Ethylene insensitive-like 1
*AtEIN3*: Ethylene insensitive 3
*AtETR1*: Ethylene response 1
ATP: Adenosine triphosphate
CaM: Calmodulin
COI1: Coronatine-insensitive1
DAMP: Damage-associated molecular pattern
LNP: Lectin–nucleotide phosphohydrolase
*DORN1*: Does Not Respond to Nucleotides1
eAde: Extracellular adenine
eAdo: Extracellular adenosine
eATP: Extracellular ATP
ECM: Extracellular matrix
ENT3: Nucleoside transporter 3
GDA1-CD39: Guanosine diphosphatase 1 cluster of differentiation 39
iATP: Intracellular ATP
JA: Jasmonic acid
JAZ1: Jasmonate ZIM-domain 1
LNP: Lectin nucleotide phosphohydrolase
NDP: Nucleoside diphosphate
NFR: Nod factor receptor
NSH3: Nucleoside hydrolase 3
NTP: Nucleoside triphosphate
NTPDase: Nucleoside triphosphate diphosphohydrolases
PAMP: Pathogen-associated molecular pattern
PCD: Programmed cell death
PSKα: Phytosulfokine α
PTI: PAMP-triggered immunity
PUP: Purine permease transporter
ROS: Reactive oxygen species
SA: Salicylic acid
TM: Transmembrane domain
